# The intersection between race/ethnicity and adverse childhood experiences and its association with depression

**DOI:** 10.1007/s00127-025-03014-y

**Published:** 2025-10-31

**Authors:** Karthik V. Rangavajhula, Ahalya Muraleedharan, Ngozi Adaralegbe, Frank Clark, Anusuiya Nagar, Nosayaba Osazuwa-Peters, Oluwole A. Babatunde, Eric Adjei Boakye

**Affiliations:** 1 Department of Chemistry and Biochemistry, College of Arts and Science, University of South Carolina, Columbia, SC USA; 2 Department of Psychiatry and Behavioral Sciences, McGovern Medical School, Houston, TX USA; 3 Prisma Health, Greer, SC USA; 4 Department of Head and Neck Surgery & Communication Sciences, Duke University School of Medicine, Durham, NC USA; 5 Duke Cancer Institute, Durham, NC USA; 6 Department of Public Health Sciences, Henry Ford Health System, One Ford Place, Detroit, MI 48202 USA; 7 Department of Otolaryngology – Head and Neck Surgery, Henry Ford Health System, Detroit, MI USA; 8 Henry Ford Health + Michigan State University Health Sciences, Detroit, MI USA; 9 Department of Epidemiology and Biostatistics, College of Human Medicine, Michigan State University, East Lansing, MI USA

**Keywords:** Adverse childhood experiences, ACE, Depression, Adults, Racial disparities, BRFSS

## Abstract

**Purpose:**

We assessed the association between number of adverse childhood experiences (ACEs) and depression among adults and explored the association by race/ethnicity.

**Methods:**

We used data from the 2020 Behavioral Risk Factor Surveillance System (BRFSS) among 127,577 adult respondents (*≥* 18 years old). The exposure was the number of ACEs classified as zero, one, two-three, and ≥ four. The outcome was a self-reported history of depression diagnosis (yes/no). Weighted multivariable logistic regression models examined the association between ACEs and depression stratified by race/ethnicity. Each model was adjusted for age, gender, smoking status, income, education, marital status, and body mass index.

**Results:**

In this sample, 36%, 23%, 21%, and 20% reported having experienced zero, one, two-three, and ≥ four ACEs, respectively. Depression was reported by 19% of survey respondents. There was a significant interaction between the number of ACEs and race/ethnicity, and depression (*p* = 0.0003), thus, analyses were stratified by race/ethnicity. Respondents who experienced ≥ 4 ACEs had higher odds of reporting depression: non-Hispanic Whites (aOR = 4.07; 95% CI: 3.55, 4.65), non-Hispanic Blacks (aOR = 3.96, 95% CI: 2.68, 5.86), or Hispanics (aOR = 7.73; 95% CI: 4.48, 13.35). Respondents with 2–3 ACEs had higher odds of reporting depression: non-Hispanic Whites (aOR: 2.41, 95% CI. 2.11– 2.76), non-Hispanic Blacks (aOR: 1.94, 95% CI. 1.19– 3.17), and Hispanics (aOR: 2.86, 95% CI. 1.64– 4.98).

**Conclusion:**

We found that individuals with two or more ACEs were more likely to report a depression diagnosis, irrespective of race/ethnicity. This finding highlights the need to monitor individuals with an increasing number of ACEs for depression.

## Introduction

Depressive disorders are the most common subset of mood disorders, significantly exacerbating the mental health burden. Our current understanding of the etiology of depression suggests that a unique balance of genetic and environmental factors contributes to onset [1–4]. In adults, depression is thought to have a stronger association with environmental exposures than genetic factors [5–7]. Among the environmental factors that can influence depression, past traumatic life experiences such as adverse childhood experiences (ACEs) are especially significant. ACEs have been found to correlate with neurological alterations on a psychological, anatomical, and molecular level [8–12]. These ACE-related effects translate to a higher likelihood of individuals engaging in certain risk behaviors and developing detrimental health conditions, including depression [13–18]. Typically, over 40–50% of patients with major depressive disorder report a history of childhood trauma [19–21]. Of these depressive disorder cases, individuals with ACE exposure were found to experience increased severity of depressive symptoms along with resistance and/or reduced adherence to treatment methodologies [22–25].

In the United States, more than 20% of adults experience depression during their lifetime and over 60% report exposure to at least one ACE [26, 27]. While impact of ACEs on the onset and prognosis of depressive disorders is substantial, there is limited understanding of how ACE exposure contributes to depression cases with differing etiologies. Such shortcomings contribute to impaired diagnoses and poorer outcomes observed in the United States to disparately affect individuals with different backgrounds [28]. Furthering our understanding of the factors that modulate this relationship between ACEs and depression can provide more reliable therapeutic and predictive algorithms to help millions of affected individuals. Race is a key factor that is known to modulate the development of various physiological and mental disorders [29, 30].

Previous studies have begun to demonstrate, separately, emerging patterns in ACE exposure between different racial groups and incidence of depression between different racial groups. While prior studies typically considered race as a covariate, further research is required to better understand how racial identity may function as a moderator on the association between ACE and the incidence of depression [31–33]. Furthermore, many of these studies are limited by small or demographically narrow samples, restricting generalizability and masking subgroup differences. In a population as racially diverse as that of the US, with many other overlapping identities, learning to tailor clinical decision making to patient’s personal characteristics is necessary to elevate outcomes [34]. To support this approach, we analyzed 2020 Behavioral Risk Factor Surveillance System (BRFSS) data to examine the association between ACE exposure and self-reported depression and whether this relationship was moderated by race/ethnicity.

## Methods

### Data source and population

Consistent with the Common Rule, this cross-sectional study of de-identified publicly available BRFSS data did not require institutional review board approval or informed consent. This analysis utilized cross-sectional data from the 2020 BRFSS. Headed by the Centers for Disease Control and Prevention (CDC)’s Population Health Surveillance Branch, the BRFSS collects health-related telephone and cellular survey data from all the states in the US and U.S. territories. The BRFSS annually surveys over 400,000 randomly selected adults on their behavioral risk factors, chronic illnesses, and use of preventive services. The questionnaire contains three components: core component, optional BRFSS modules, and state-added questions. The core component encompasses a set of basic questions inquiring about demographics, health behaviors, perceptions, etc. Secondly, the optional modules contain state-selected questions tailored to focus on a topic (e.g. HPV vaccination, ACE) which varies by state. The third set of questions is developed by individual states. More in-depth information about the BFRSS survey methodology has been published by CDC [35].

As part of the second questionnaire component, CDC included a module on ACE questions. In 2020, the following 28 states included the ACE data in their BFRSS survey: AL, AZ, CA, DC, FL, GA, HI, ID, IA, KS, KY, MD, MA, MS, MO, MT, NV, NJ, ND, OK, RI, SC, SD, TX, UT, VA, WI, WY. The focus of this study was the general adult population aged 18 years or older with usable responses to the ACE survey components (*n* = 127,577).

### Outcome measure

The outcome variable was a self-reported history of depression, which was assessed with the question: “Has a doctor, nurse, or other health professionals ever told you that you had a depressive disorder (including depression, major depression, dysthymia, or minor depression)?”. The respondents who answered “yes” to the question were reported to have a history of depression (called depression thereafter).

### Exposures

The exposures were the number of ACEs experienced before the age of 18 and race/ethnicity. ACEs comprised 11 questions that determined exposure to the following three types of abuse (physical, emotional, and sexual) and five types of household challenges (substance abuse, incarceration/mental illness, parental divorce, and intimate partner violence) [35]. The ACE questions are grouped into these categories: mental health challenges in the household, substance abuse in the household, incarcerated household members, violence by adults in the household, sexual harassment by older family members, and parental separation [35]. The presence of each ACE category received a score of 1. Otherwise, it received a score of 0. The categories were then added to produce an ACE score ranging from 0 to 11, with a higher score indicating a higher number of ACEs. The ACE scores were classified into four categories: zero, one, two-three, and ≥ 4 based on previous literature [36, 37]. Race/ethnicity categories included non-Hispanic White, non-Hispanic Black, Hispanic, and non-Hispanic Other. Race/ethnicity was used as a moderator and was classified as non-Hispanic White, non-Hispanic Black, Hispanic, or non-Hispanic Other.

### Covariates

The covariates included in the analyses were: age (18–39, 40–64, ≥ 65), sex (female, male), marital status (married/partnered, divorced/separated/widowed, or never married), education level (college graduate, some college, or < High School (HS)/HS graduate), income level (≥$50,000, $25,000 to 49,999, <$25,000, or missing), body mass index (BMI) (normal, overweight, or obese), smoking status (never, former, or current). For BMI, within 18.5 to < 25 kg/m2 was defined as normal.

### Statistical analyses

All analyses were performed using survey-specific procedures, which incorporate survey sampling weights to account for the complex sampling design used in the BRFSS and to provide representative estimates of the U.S. population. Respondents’ characteristics overall and stratified by number of ACEs were used to describe the study sample. Differences in respondents’ characteristics were compared with the number of ACEs using Chi-square. To test for the interaction between race/ethnicity and ACE categories and the association with depression, a multivariable logistic regression model was performed, adjusting for covariates (age, sex, smoking status, income, education, marital status, and body mass index). The interaction between the number of ACEs and race/ethnicity, and depression was statistically significant (*P* < 0.05). Given the statistically significant interaction, four separate multivariable logistic regression models (one for each race/ethnicity category) were used to examine the association between the number of ACEs and depression, adjusting for the same covariates mentioned above. All tests were 2-sided, with *P* < 0.05 considered statistically significant. All analyses were conducted in SAS version 9.4 (SAS Institute).

## Results

A total of 127,577 respondents were included in the study, of whom 19.1% (*n* = 24,305) reported being diagnosed with depression (Table 1). Approximately 39% (*n* = 50,128) of respondents experienced no ACEs, 22.6% (*n* = 28,855) experienced one ACE, 20.7% (*n* = 26,446) experienced 2–3 ACEs, and 17.4% (22,148) experienced ≥ four ACEs. Overall, most respondents were female (52.4%), Non-Hispanic White (60.6%), married or partnered (55.4%), never smoked (60.3%), and BMI was equally distributed: normal (34.4%), overweight (34.3%), and obese (34.3%). In the crude analysis, all the variables were significantly associated with the ACE scores, with a p-value of < 0.0001.

**Table 1:** Sample characteristics overall and stratified by number of ACEs, 2020 behavioral risk factor surveillance system (*n* = 127,577)

	Frequency (weighted percent)					*P*-value^&^
	Overall (*n* = 127,577; 100%)	No ACEs (*n* = 50,128; 39.3%)	One ACE (*n* = 28,855; 22.6%)	2–3 ACEs (*n* = 26,446; 20.7%)	≥ 4 ACEs (*n* = 22,148; 17.4%)	
*Depression*						
Yes	24,305 (19.1)					
No	103,272 (80.9)					
*Race/ethnicity*						
Non-Hispanic White	93,413 (60.6)	37,975 (60.6)	20,818 (58.7)	19,166 (61.7)	15,454 (61.3)	< 0.0001
Non-Hispanic Black	11,290 (13.7)	4071 (12.3)	2971 (15.7)	19,166 (61.7)	1872 (13.3)	
Hispanic	9890 (17.8)	3424 (18.2)	2240 (18.1)	2122 (16.8)	2104 (17.9)	
Non-Hispanic Other	12,984 (7.9)	4658 (8.9)	2240 (18.1)	2782 (7.1)	2718 (7.5)	
*Age*						
18–39	28,740 (35.5)	8148 (10.3)	6253 (35.1)	6568 (37.3)	7772 (46.9)	< 0.0001
40–64	49,898 (40.7)	17,627 (14.1)	11,080 (40.4)	11,091 (41.9)	10,100 (42.6)	
≥ 65	47,114 (23.7)	23,386 (11.6)	11,115 (24.5)	8509 (20.8)	4104 (10.5)	
*Gender*						
Female	69,998 (52.4)	27,425 (52.6)	14,938 (48.7)	14,160 (49.9)	13,475 (58.9)	< 0.0001
Male	57,579 (47.6)	22,703 (47.4)	13,917 (51.3)	12,286 (50.1)	8673 (41.1)	
*Marital status*						
Married/partnered	70,872 (55.4)	29,503 (59.7)	16,046 (55.4)	14,282 (53.3)	11,041 (49.8)	< 0.0001
Divorced/separated/widowed	34,618 (21.4)	13,755 (21.1)	7699 (20.8)	7024 (22.1)	6140 (22.1)	
Never married	21,280 (23.2)	6492 (19.2)	4945 (23.8)	4988 (24.6)	4855 (28.1)	
*Education level*						
College graduate	46,677 (24.7)	20,009 (30.0)	10,755 (25.7)	9533 (24.7)	6380 (19.0)	< 0.0001
Some college	37,378 (32.4)	13,611 (30.1)	8222 (34.9)	8025 (34.9)	7520 (35.8)	
< High School/HS graduate	43,200 (41.9)	16,336 (39.9)	9809 (43.4)	8838 (40.4)	8217 (46.2)	
*Income level*						
>=$50,000	55,294 (41.8)	22,161 (42.4)	12,741 (42.5)	11,839 (43.4)	8553 (38.3)	< 0.0001
$25,000 to 49,999	25,707 (19.4)	9542 (17.5)	5818 (19.4)	5545 (20.5)	4802 (21.6)	
<$25,000	25,430 (21.8)	8381 (19.5)	5486 (20.8)	5399 (21.7)	6164 (27.4)	
Missing	21,146 (17.0)	10,044 (20.6)	4810 (17.3)	3663 (14.4)	2629 (12.7)	
*BMI*						
Normal	37,582 (34.4)	15,474 (32.7)	8400 (31.6)	7561 (30.3)	6147 (30.2)	< 0.0001
Overweight	42,343 (34.3)	17,200 (36.6)	9834 (34.2)	8690 (33.4)	6619 (31.3)	
Obese	39,590 (34.3)	13,727 (30.7)	8843 (34.2)	8733 (36.3)	8287 (38.5)	
*Smoking status*						
Never	75,206 (60.3)	33,480 (68.4)	17,333 (62.3)	14,547 (56.4)	9846 (47.7)	< 0.0001
Former	33,995 (24.2)	11,999 (22.0)	7782 (23.4)	7613 (26.2)	6601 (27.0)	
Current	17,676 (15.5)	4332 (9.6)	3577 (14.3)	4157 (17.4)	5610 (25.3)	

ACE = Adverse childhood experience

^&^*P*-value based on the chi-square test

In the multivariable analysis examining the association between number of ACEs and depression, a dose-response association was observed. Respondents who had experienced one ACE (adjusted odds ratio [aOR] = 1.57; (95% confidence interval [CI]: 1.41, 1.74), 2–3 ACEs (aOR = 2.43; 95% CI: 2.19, 2.70), or ≥ 4 ACEs (aOR = 4.74; 95% CI: 4.27, 5.25) had higher odds of reporting being diagnosed with depression compared to those that had not experienced any ACE as a child (Table 2). With regards to race/ethnicity, compared to non-Hispanic Whites, non-Hispanic Blacks (aOR = 0.52; 95% CI: 0.46, 0.59), Hispanic (aOR = 0.58; 95% CI: 0.50, 0.67) and non-Hispanic Others (aOR = 0.74; 95% CI: 0.65, 0.84) had lower odds of reporting being diagnosed with depression.

**Table 2:** Associations between number of ACEs, race/ethnicity and depression, 2020 behavioral risk factor surveillance system (*n* = 127,577)

	Adjusted odds ratio (99% Confidence Interval)
*Number of ACEs*	
No ACEs	Reference
One ACE	1.57 (1.41, 1.74)
2–3 ACEs	2.43 (2.19, 2.70)
≥ 4 ACEs	4.74 (4.27, 5.25)
*Race/ethnicity*	
Non-Hispanic White	Reference
Non-Hispanic Black	0.52 (0.46, 0.59)
Hispanic	0.58 (0.50, 0.67)
Non-Hispanic Other	0.74 (0.65, 0.84)

ACE = Adverse childhood experience

Models adjusted for age, gender, marital status, education level, income level, BMI, smoking status

Table 3 shows multivariable regression models for each race/ethnicity. Among non-Hispanic Whites, higher odds of reporting being diagnosed with depression was reported by those who had experienced one ACE (aOR = 1.54; (95% CI: 1.33, 1.77), 2–3 ACEs (aOR = 2.41; 95% CI: 2.11, 2.76), or ≥ 4 ACEs (aOR = 4.07; 95% CI: 3.55, 4.65) compared to those that had not experienced any ACE as a child. Similarly, non-Hispanic Black respondents had higher odds of reporting being diagnosed with depression if they experienced one ACE (aOR = 1.44; 95% CI: 0.91, 2.26), 2–3 ACEs (aOR = 1.94; 95% CI: 1.19, 3.17), or ≥ 4 ACEs (aOR = 3.96; 95% CI: 2.68, 5.86) compared to those that had not experienced any ACE as a child. The pattern stayed consistent with the Hispanic and Non-Hispanic Other respondents. Hispanic respondents who experienced 2–3 ACEs (aOR = 2.86; 95% CI: 1.64, 4.98) or ≥ 4 ACEs (aOR = 7.73; 95% CI: 4.48, 13.35) had a higher likelihood of reporting being diagnosed with depression compared to those who had not experienced any ACE as a child. Among non-Hispanic Others, higher odds of reporting being diagnosed with depression was reported by those who had experienced 2–3 ACEs (aOR = 2.65; 95% CI: 1.54, 4.56), or ≥ 4 ACEs (aOR = 7.73; 95% CI: 4.58, 13.03) compared to those that had not experienced any ACE as a child.

**Table 3:** Four multivariable logistic models (one for each race/ethnicity) estimating associations between number of aces and depression, 2020 behavioral risk factor surveillance system (*n* = 127,577)

	Adjusted odds ratio (99% Confidence Interval)			
	Non-Hispanic White (*n* = 93,413)	Non-Hispanic Black (*n* = 11,290)	Hispanic (*n* = 9,890)	Non-Hispanic Other (*n* = 12,984)
*Number of ACEs*				
No ACEs	Ref	Ref	Ref	Ref
One ACE	1.54 (1.33, 1.77)	1.44 (0.91, 2.26)	1.72 (0.96, 3.08)	1.70 (0.93, 3.08)
2–3 ACEs	2.41 (2.11, 2.76)	1.94 (1.19, 3.17)	2.86 (1.64, 4.98)	2.65 (1.54, 4.56)
≥ 4 ACEs	4.07 (3.55, 4.65)	3.96 (2.68, 5.86)	7.73 (4.48, 13.35)	7.73 (4.58, 13.04)
*Age*				
18–39	Ref	Ref	Ref	Ref
40–64	0.85 (0.75, 0.96)	0.93 (0.64, 1.35)	0.94 (0.63, 1.42)	0.83 (0.57, 1.22)
≥ 65	0.57 (0.49, 0.66)	0.66 (0.39, 1.11)	0.98 (0.55, 1.76)	0.86 (0.54, 1.38)
*Sex*				
Male	Ref	Ref	Ref	Ref
Female	2.39 (2.16, 2.64)	2.16 (1.59, 2.94)	1.91 (1.32, 2.77)	1.95 (1.42, 2.67)
*Marital status*				
Married/partnered	Ref	Ref	Ref	Ref
Divorced/separated/widowed	1.31 (1.17, 1.48)	1.50 (1.02, 2.21)	2.20 (1.44, 3.38)	1.17 (0.80, 1.72)
Never married	1.37 (1.18, 1.59)	1.35 (0.87, 2.08)	1.77 (1.12, 2.80)	1.12 (0.73, 1.70)
*Education level*				
College graduate	Ref	Ref	Ref	Ref
Some college	1.01 (0.90, 1.14)	0.97 (0.63, 1.49)	1.23 (0.73, 2.05)	1.16 (0.78, 1.72)
< High School/HS graduate	0.83 (0.73, 0.94)	0.80 (0.46, 1.41)	1.06 (0.66, 1.71)	0.95 (0.62, 1.50)
*Income level*				
>=$50,000	Ref	Ref	Ref	Ref
$25,000 to 49,999	1.32 (1.15, 1.51)	0.85 (0.48, 1.50)	0.86 (0.51, 1.44)	1.50 (0.99, 2.29)
<$25,000	1.91 (1.66, 2.21)	1.58 (0.85, 2.95)	1.16 (0.69, 1.95)	1.84 (1.22, 2.76)
*BMI*				
Normal	Ref	Ref	Ref	Ref
Overweight	1.19 (1.05, 1.35)	0.87 (0.58, 1.32)	0.98 (0.62, 1.55)	1.03 (0.93, 1.95)
Obese	1.72 (1.53, 1.94)	0.90 (0.59, 1.37)	0.97 (0.63, 1.45)	1.89 (1.23, 2.91)
*Smoking status*				
Never	Ref	Ref	Ref	Ref
Former	1.30 (1.16, 1.46)	2.14 (1.46, 3.16)	1.72 (1.08, 2.75)	1.35 (0.93, 1.95)
Current	1.57 (1.37, 1.79)	2.18 (1.49, 3.18)	1.62 (0.98, 2.69)	2.39 (1.62, 3.50)

ACE = Adverse childhood experience

All models adjusted for age, gender, marital status, education level, income level, BMI, smoking status

## Discussion

In this study, we examined whether the race/ethnicity of an individual modulates the relationship between ACE exposure and self-reported depression, based on 127,577 responses to the 2020 multi-layered BRFSS survey. We found that respondents reporting ≥ 2 ACEs exposures as a child have higher odds of being diagnosed with depression, across all racial/ethnic groups. Merrick et al. noted meaningful differences in the number of ACEs among different racial groups using the 2011–2014 BRFSS data [38]. Giano et al. also used BRFSS data from 2009 to 2017 and noted the disproportionately higher number of ACEs amongst multiracial individuals [39, 40]. However, none of the studies investigated the relationship between ACE exposure and depression diagnosis, and how race/ethnicity modulates the relationship. Our findings could help identify target groups for intervention or provide a level of clarity to the challenges in classifying difficult-to-treat depression, as outlined by Rush et al. [41]. While our findings indicate racial differences in the strength of the association between ACEs and depression, the exact magnitude of these differences between groups and their underlying mechanisms remain insufficiently understood. Existing hypotheses are underdeveloped and require more nuanced approaches to fully capture this complex relationship. Nevertheless, our analysis provides valuable documentation of population-level differences, underscoring the need for longitudinal, clinically validated, and culturally contextualized research.

### Potential biological bases of ACE-depression link

Our finding that a higher number of ACEs is associated with depression, irrespective of race/ethnicity, could be explained by several factors. ACE exposure involves dysregulation of the hypothalamic-pituitary-adrenal (HPA) axis, which is crucial in managing stress response in the body [42–44]. This can manifest as alterations in cortisol levels, which can result in a series of dysregulated physiological responses induced by increased allostatic load [45, 46]. Elevation in stress factors can result in chronic systemic inflammation, which is known to be biologically linked to mood disorders via effects on the gut microbiome and triglyceride metabolism [47]. Depression onset related to increased cortisol levels is classically linked to ACE and plays a substantial role in translating the exposure effects into health complications [48]. Compared to their white counterparts, minority children are noted to have higher daily cortisol exposure, which can compound throughout the child’s life to induce health issues [49].

Reduced diurnal cortisol response is thought to be an effect of allostatic overload, as the body reacts to minimize stress reactivity. However, this response pattern is often associated with more severe depressive symptomatology, treatment resistance, and higher sensitivity to pro-inflammatory factors [50]. It should be noted that some level of this decrease in free cortisol levels is thought to be linked to alternative hormonal pathways [48]. However, they reflect an interesting trend in the effects of ACE exposure. Considering the pattern of adult hypocortisolism seen in minority groups is also observed in individuals with ≥ 3 ACEs, it is possible the racialized stress experiences unique to minority populations (which often can fall beyond the BRFSS classification of ACE exposure) demonstrate comparable levels of influence on HPA dysregulation as high levels of ACE exposure [28]. This biological effect of cumulative stress exposure is consistent with the increase in odds of depression with elevated number of ACE exposures we report from the BRFSS.

### Potential social bases of ACE-depression link

Discrimination is known to co-occur with ACEs in racial minority groups and can compound the effects of ACE exposure [51]. Biologically, racial discrimination is associated with many of the same pathways of effect as ACEs, such as dysregulation of the HPA axis, elevation in inflammatory cytokines, and epigenetic modifications [52]. While the biological and social effects of discrimination or ACEs can synergize to directly heighten the physiological imbalance, the social effects can also indirectly inhibit potential protective factors [53]. Protective factors derived from an individual’s support systems are known to generate resiliency against the development of mental health disorders. Among minority groups, the strongest protective effect is thought to be conferred by family cohesion, parental guidance, and broad support networks involving both peers and adult figures [54]. However, discriminatory barriers to community engagement and ACE-induced disruption of familial cohesion can impair the development of the social support characteristic of the aforementioned protective factors [54, 55]. Previous studies among immigrant Hispanic populations observed that the uprooting and lack of socioeconomic security can degrade family cohesion and increase cohesion and a corresponding psychological distress [56]. In some circumstances, family cohesion can be linked with higher distress. This is in line with our findings. On the contrary, more socially integrated minority groups like Asian populations in the US report strengthened family protective effect [57]. In instances when both ACE exposure and discrimination affect a child’s development, the direct loss of family and community structure, and the indirect impairment of socialization, academic engagement, and cultural identity, could potentially synergize to dramatically increase risk of mood disorder onset.

African-American populations are reported to have very robust familial support systems that cross generational and geographic gaps [58, 59]. This network offers instrumental support against patterns of socioeconomic challenges and discrimination that also affect this group. These patterns may explain the potential weaker correlation of ACE exposure and incidence of depression in Black populations that we found in this study, despite a high level of ACE exposure. At the same time, the previously mentioned contingency on access to a diagnosis of depression may skew our results, especially with Black populations. Given low rates of initiating treatment, high distrust of healthcare providers, and familial stigma that inhibits help-seeking behavior, the number of Black respondents with depression could very well be underrepresented [60–63]. In order to accurately characterize these unique factors, more conscious survey design may be required.

### Study implications

This study targets a topic that has historically lacked focus in comprehensive studies. Cavanaugh and Nelson found that, of the few studies on depression in Black populations, many had limited participants and did not cover large geographical areas [43]. Studies focused on Hispanic populations also suffer from the same poor generalization because of a lack of diversity among the study population [64]. Our work, pulling from the BRFSS, provides a more comprehensive and representative overview of different populations across the nation, leading to increased statistical power to better assess the mental health crisis across the United States as it relates to ACEs, race/ethnicity, and depression. This knowledge can inform approaches to improve health at the individual and community levels by highlighting relevant risk factors among certain populations. Further depth of analysis on this topic and subsequent clinician education will be required to maximize the potential benefit that may be incurred by millions who are experiencing or are at risk of developing depressive symptoms.

### Limitations

Our study has limitations. First, both the outcome and exposure were self-reported, and could be susceptible to recall and social desirability biases, therefore, future studies using objective measures are needed. In addition, the outcome was measured—depression—was using a single item question which may underrepresent individuals who have not had access to formal mental health treatment. However, previous studies have used the exact same question to access depression; future research should use other validated questionnaires. Second, we are unable to determine when in the respondents’ lifetime the depression diagnosis occurred or if the disease state persists to the time of response, limiting our analytical capacity regarding the chronicity of depression. Third, our sample was drawn from only several states that included the ACE modules; thus, it should not be considered nationally representative because of state-to-state variations. Fourth, the study was cross-sectional, thus, no causal inference could be made.

## Conclusions

In summary, respondents with a history of ACEs were more likely to report depression compared to those with no ACE exposure, irrespective of their racial/ethnic group. These findings emphasize the complex and multifaceted relationship between childhood adversity and adult mental health outcomes. By elucidating the impact of ACEs on depression, this research provides valuable insights into the enduring psychological consequences of early life adversity. Moreover, it highlights the need for targeted interventions and culturally sensitive support systems to address the mental health needs of individuals with ACE histories. Further investigation is warranted to refine these approaches and enhance their effectiveness in mitigating long-term mental health challenges.

## Data Availability

The data used in this study are publicly available and can be accessed from the BRFSS website at [https://www.cdc.gov/brfss/data\_documentation/index.htm](https:/www.cdc.gov/brfss/data_documentation/index.htm).
